# Reproductive factors, tumor estrogen receptor status and contralateral breast cancer risk: results from the WECARE study

**DOI:** 10.1186/s40064-015-1642-y

**Published:** 2015-12-30

**Authors:** Julia S. Sisti, Jonine L. Bernstein, Charles F. Lynch, Anne S. Reiner, Lene Mellemkjaer, Jennifer D. Brooks, Julia A. Knight, Leslie Bernstein, Kathleen E. Malone, Meghan Woods, Xiaolin Liang, Esther M. John, the WECARE Study Collaborative Group

**Affiliations:** Memorial Sloan Kettering Cancer Center, 485 Lexington Avenue, 2nd Floor, New York, NY 10017 USA; University of Iowa, Iowa City, IA USA; Danish Cancer Society Research Center, Copenhagen, Denmark; University of Toronto, Dalla Lana School of Public Health, Toronto, Canada; Lunenfeld-Tanenbaum Research Institute, Mount Sinai Hospital, Toronto, Canada; Beckman Research Institute, City of Hope National Medical Center, Duarte, CA USA; Fred Hutchinson Cancer Research Center, Seattle, WA USA; Cancer Prevention Institute of California, Fremont, CA USA; Department of Health Research and Policy (Epidemiology) and Stanford Cancer Institute, Stanford University School of Medicine, Stanford, CA USA

**Keywords:** Contralateral breast cancer, Reproductive risk factors, Breastfeeding, Parity, Estrogen receptor

## Abstract

Several reproductive factors are known to be associated with risk of breast cancer; however, relationships between these factors with risk of second primary asynchronous contralateral breast cancer (CBC) have not been widely studied. The Women’s Environmental, Cancer, and Radiation Epidemiology (WECARE) Study is a population-based case-control study of 1521 CBC cases and 2212 individually matched controls with unilateral breast cancer. Using multivariable conditional logistic regression models, we examined associations between reproductive factors and CBC risk, and whether associations differed by estrogen receptor (ER) status and menopausal status of the first breast cancer. Older age at menarche was inversely associated with CBC risk (≥14 vs. ≤11 years risk ratio (RR) = 0.82, 95 % confidence interval (CI) 0.65–1.03, *P*_trend_ = 0.02). Among parous women, an increasing number of full-term pregnancies (FTP) was inversely associated with risk (≥4 vs. 1 FTP RR = 0.60, 95 % CI 0.41–0.88, *P*_trend_ = 0.005). Ever breast-feeding was inversely associated with CBC risk only among women with ER-negative first tumors (ever vs. never breast-fed RR = 0.69, 95 % CI 0.48–1.00, *P*_heterogeneity_ = 0.05). Older age at first FTP was inversely associated with CBC risk among women with ER-negative first tumors (≥30 vs. <20 years old RR = 0.66, 95 % CI 0.35–1.27, *P*_trend_ = 0.03), but suggestively positively associated with risk among women with ER-positive first tumors (*P*_heterogeneity_ = 0.03). Young age at menarche and low parity, both risk factors for first primary breast cancer, were also associated with overall CBC risk. Reductions in risk associated with breast-feeding were limited to women with ER-negative first tumors, who are at higher CBC risk than women with ER-positive primaries.

## Background

Reproductive factors, including menstrual and reproductive history, are well-established predictors of breast cancer risk. Young age at menarche and late age at menopause, representing an increased number of lifetime menstrual cycles, are associated with increased risk (Collaborative Group on Hormonal Factors in Breast Cancer 2012). Parity is associated with decreased risk, though a protective effect is observed only among women with young ages at first birth; women with a first birth occurring after age 35 years tend to be at a higher lifetime risk relative to nulliparous women (Rosner et al. 1994; Trichopoulos et al. 1983). Substantial evidence suggests that associations between reproductive factors and breast cancer vary by tumor subtypes defined by hormone receptor status and molecular subtype (Anderson et al. 2014). For example, inverse associations with young age at first birth and higher parity are stronger for estrogen receptor-positive (ER-positive) compared to estrogen receptor-negative (ER-negative) tumors (Althuis et al. 2004; Ma et al. 2006; Ritte et al. 2013; Yang et al. 2011). Conversely, the protective effect of breast-feeding appears to be stronger for, or limited to, ER-negative subtypes (Ma et al. 2006; Ambrosone et al. 2014; Gaudet et al. 2011; Li et al. 2013; Millikan et al. 2008; Tamimi et al. 2012).

Among women diagnosed with breast cancer, the risk of developing a second primary asynchronous contralateral breast cancer (CBC) is greater than the risk of developing a primary breast cancer in the general population (Curtis RE et al. 1973). First and second primary breast tumors have been shown to share some risk factors, including mutations in *BRCA1* and *BRCA2* (Graeser et al. 2009; Metcalfe et al. 2004), family history of breast cancer (Bernstein et al. 1992; Hemminki et al. 2007), and obesity (Druesne-Pecollo et al. 2012). Reproductive factors have been implicated in the etiology of CBC, though associations have been less consistent than those observed for first primary breast cancers. In the first phase of the Women’s Environmental, Cancer, and Radiation (WECARE) Study, a population-based case-control study of cases with CBC and controls with unilateral breast cancer (UBC), we observed an inverse association between number of full-term pregnancies and CBC risk, and a positive association with young age at menarche (Largent et al. 2007). Some prior studies have similarly observed a reduced risk of CBC with parity (Bernstein et al. 1992; Ricceri et al. 2015; Storm et al. 1992) and younger age at first birth (Vaittinen and Hemminki 2000), though others, potentially limited by small sample sizes, have reported null associations with these and other reproductive factors (Boice et al. 1992; Cook et al. 1996; Horn and Thompson 1988; Li et al. 2003). To date, no studies of CBC have investigated whether these associations are modified by estrogen receptor (ER) status.

In the present study, we sought to clarify the associations between reproductive factors and CBC risk in the WECARE Study population, which recently completed its second phase. In addition to the 708 matched case-control triplets included in our previous analysis (Largent et al. 2007), the current analysis includes an additional 813 matched case-control pairs, giving us substantially increased power to examine these relationships. Our large and expanded sample size enabled us to examine potential heterogeneity by ER status and menopausal status of the first primary tumor.

## Methods

### Study population

The WECARE Study is a multicenter, population-based case-control study of cases with asynchronous CBC and individually matched controls with UBC. The study design of the first phase (WECARE I) has been described in detail elsewhere (Bernstein et al. 2004); the second phase (WECARE II) employed a nearly identical approach [Langballe et al, submitted]. Briefly, participants in each phase were identified through eight population-based cancer registries, including six in the United States that contribute to the National Cancer Institute’s Surveillance, Epidemiology, and End Results (SEER) program: Los Angeles County Cancer Surveillance Program; Cancer Surveillance System of the Fred Hutchinson Cancer Research Center (Seattle region, WA); State Health Registry of Iowa; Cancer Surveillance Program of Orange County/San Diego-Imperial Organization for Cancer Control (Orange County/San Diego, CA); the Greater Bay Area Cancer Registry (San Francisco Bay Area region and Santa Clara region, CA); and the Sacramento and Sierra Center Registry (Sacramento region, CA). Participants were additionally identified using the Ontario Cancer Registry (Canada) and the Danish Breast Cancer Cooperative Group Registry, supplemented by data from the Danish Cancer Registry. The study protocol was approved by the institutional review boards at each study site and by the Ethics Committee System in Denmark.

Eligible cases were women who: (1) were diagnosed between 1985 and 2009 with a first invasive breast cancer that did not spread beyond regional lymph nodes at diagnosis and a second contralateral primary breast cancer at least 1 year after the first breast cancer diagnosis (reference date); (2) were younger than 55 years at first diagnosis; (3) had no previous or intervening cancer diagnosis except non-melanoma skin cancer or cervical carcinoma in situ; (4) were alive at the time of contact and able to provide informed consent to complete the interview and provide a biospecimen; and (5) resided in the same cancer registry reporting region for both diagnoses. Eligible controls with an intact contralateral breast were identified using the same eligibility criteria, and individually matched to cases (1:2 in WECARE I; 1:1 in WECARE II) on the following criteria: year of birth (5-year strata), year of diagnosis (4-year strata), cancer registry region, and race/ethnicity. For each control, reference date was created by adding the at-risk period of her matched case to the date of her breast cancer diagnosis. WECARE I cases and controls were counter-matched on radiation exposure, such that two members of the case-control triad had received radiation therapy for their first breast cancer. In total, 2354 CBC cases and 3599 UBC controls met eligibility criteria and were approached for inclusion in the study. Of those eligible women, 1521 cases (64.6 %) and 2212 (61.5 %) controls completed the interview, provided a biospecimen (blood or saliva), and provided written informed consent.

### Data collection

Study participants were interviewed by telephone using a structured questionnaire aimed at evaluating known or suspected breast cancer risk factors, including personal demographics, medical history, menstrual and reproductive history, family history of cancer, use of hormones, smoking, and alcohol intake. Risk factor status was assessed during the period prior to first diagnosis, as well as between first diagnosis and reference date (i.e., the at-risk period for CBC). Reproductive risk factors that were assessed included: age at menarche, number of pregnancies, duration and outcome of each pregnancy, date each pregnancy ended, duration of breast-feeding for each live birth, menopausal status, age at menopause and reason for menopause. A full-term pregnancy (FTP) was defined as a pregnancy that resulted in a stillbirth or at least one live birth. Breast-feeding duration was calculated by summing months of breast-feeding duration for each live birth. Detailed data on treatment and tumor characteristics, including ER and progesterone receptor (PR) status, were obtained directly from cancer registry records or by abstracting medical records, including pathology and surgical reports, radiation oncology clinic notes, and systemic adjuvant treatment data. Self-reported treatment data were used for participants with missing information in their medical records (chemotherapy, 4 %; hormonal therapy, 5 %).

### Statistical analysis

Multivariable-adjusted risk ratios (RRs) and corresponding 95 % confidence intervals (CIs) were estimated by fitting conditional logistic regression models to the combined WECARE I and WECARE II data. We evaluated the following reproductive risk factors among all women: age at menarche (≤11, 12, 13, ≥14 years), parity (parous vs. nulliparous), menopausal status (2 years prior to first breast cancer diagnosis) and age at menopause (premenopausal, postmenopausal/≤45 years, postmenopausal/>45 years). In order to reduce the potential misclassification of menopausal status due to treatment-induced menstrual irregularities, we considered women premenopausal at first diagnosis if they reported menstruating or being pregnant in the 2 years before diagnosis. We examined number of FTP (1, 2, 3, ≥4), age at first FTP (< 20, 20–24, 25–29, ≥30 years), time between menarche and first FTP (<10, 10–14, 15–19, ≥20 years), time since last FTP (<5, 5 to <10, 10 to <15, 15 to <20, ≥20 years) and duration of breast-feeding (never, ≤6, 7–12, 13–24, ≥25 months). We also evaluated whether time since last birth was associated with CBC risk, using the method outlined by Heuch et al. (1999) to avoid linear dependence between attained age, age at birth and time since birth among uniparous women. Briefly, nulliparous women were assigned to the referent categories for age at first FTP and time since last FTP and included in models with parous women; effects were estimated among parous women only by adding an indicator for parity (yes, no) to the model. Multivariable models were adjusted for the following known and suspected CBC risk factors: age at first breast cancer diagnosis (continuous), first-degree family history of breast cancer (yes, no, adopted/unknown), lobular histology of first breast cancer (yes, no, unknown), and receipt of hormonal treatment (yes, no, unknown), radiation therapy (yes, no, unknown) and/or chemotherapy (yes, no, unknown) for first diagnosis. Further adjustment for type of chemotherapy (i.e., taxanes, anthracycline-based regimens, cyclophosphamide with methotrexate and fluorouracil, or other chemotherapeutics) did not appreciably change results, and this covariate was not retained in final models. Models were additionally mutually adjusted for the reproductive factors of interest, with the exception of time since last FTP, which we hypothesized may lie on the causal pathway between pregnancy-related variables and CBC risk.

Time-varying reproductive factors, including number and age at FTP and breast-feeding, were evaluated at the time of first diagnosis as well as at the reference date. Analyses produced similar results and, with the exception of menopausal status, estimates are shown for reproductive variables as of the reference date.

In additional analyses, we examined whether associations between reproductive factors and CBC risk differed according to ER status of the first primary tumor, menopausal status at first diagnosis, and time since first diagnosis. Because PR status was unknown for approximately 25 % of first primary tumors, we did not examine whether associations differed by joint ER and PR status. However, among tumors with data on both markers, approximately 90 % of ER-positive first tumors were also PR-positive; similarly, about 90 % of ER-negative first tumors were also PR-negative. Likelihood ratio tests were used to assess heterogeneity by potential effect modifiers. Analyses were conducted using SAS v. 9.4 (SAS Institute, Cary, NC).

## Results

Characteristics of the 1521 CBC cases and 2212 UBC controls included in our analysis are shown in Table 1. Median age at first diagnosis was 46 years; among cases, the median time to CBC diagnosis was 6.3 years. Data on ER status of the first primary tumor were available for approximately 83 % of participants (83 % for cases, 82 % for controls); among women with available ER status, 67 % had ER-positive first tumors. Approximately 80 % of participants reported at least one FTP at the time of first diagnosis, with a median age at first FTP of 24 years.

**Table 1:** Characteristics of contralateral breast cancer (CBC) cases and unilateral breast cancer (UBC) controls from the WECARE Study population

Variable	CBC cases N = 1521		UBC controls N = 2212	
	Median	Range	Median	Range
Age at first diagnosis (years)	46	24–55	46	23–55
Age at reference date (years)	53	27–73	52	27–71
Length of at-risk period (years)^a^	6.3	1.0–19.8	5.5	1.0–19.8
	N (%)		N (%)	
Study center				
Iowa	201 (13)		314 (14)	
California sites	658 (43)		967 (44)	
Seattle	224 (15)		317 (14)	
Denmark	279 (18)		457 (21)	
Ontario	159 (10)		157 (7)	
Year of diagnosis				
1985–1988	238 (16)		467 (21)	
1989–1992	415 (27)		647 (29)	
1993–1996	427 (28)		632 (29)	
1997–2008	441 (29)		466 (21)	
First degree family history of breast cancer				
No	1004 (66)		1706 (77)	
Yes	497 (33)		466 (21)	
Adopted/unknown	20 (1)		40 (2)	
Histology of first diagnosis				
Lobular	179 (12)		223 (10)	
Other	1338 (88)		1986 (90)	
Unknown	4 (0)		3 (0)	
Stage of first diagnosis				
Local	1061 (70)		1442 (65)	
Regional	448 (29)		759 (34)	
Unknown	12 (1)		11 (1)	
Estrogen receptor (ER) status of first diagnosis^b^				
Positive	797 (52)		1254 (57)	
Negative	467 (31)		561 (25)	
Other	257 (17)		397 (18)	
Progesterone receptor (PR) status of first diagnosis^b^				
Positive	687 (45)		1083 (49)	
Negative	442 (29)		549 (25)	
Other	392 (26)		580 (26)	
Chemotherapy				
No	699 (46)		923 (42)	
Yes	822 (54)		1289 (58)	
Radiation treatment				
No	641 (42)		525 (24)	
Yes	880 (58)		1686 (76)	
Unknown	0 (0)		1 (0)	
Hormone treatment				
No	964 (63)		1270 (57)	
Yes	557 (37)		940 (43)	
Unknown	0 (0)		2 (0)	

^a^Beginning at least 1 year after first diagnosis extending to the reference date (date of second diagnosis in cases)

^b^Refers to receptor status of the first primary breast cancer. The ‘Other’ category consists of women where no lab test was given, the test was given and the results are unknown or the test was given and the results were borderline

Women who reported age at menarche of 14 years or older had an 18 % lower risk of CBC compared to those who had their first menses at age 11 years or younger (RR = 0.82, 95 % CI 0.65–1.03, *P*_trend_ = 0.02) (Table 2). Women with at least one FTP at the time of first diagnosis were not at a reduced risk of CBC compared to nulliparous women, and a pregnancy occurring after first diagnosis was not associated with risk. Among parous women, increasing parity was inversely associated with risk (≥4 vs. 1 FTP RR = 0.60, 95 % CI 0.41–0.88, *P*_trend_ = 0.005) but there was no clear trend between increasing age at first FTP and CBC risk (*P*_trend_ = 0.82). A long interval between menarche and first FTP was suggestively associated with increased CBC risk, though no statistically significant trend was observed (≥20 vs. <10 year interval RR = 1.33, 95 % CI 0.94–1.88, *P*_trend_ = 0.12). No clear trends between CBC risk and either breast-feeding duration or time since last birth were observed among parous women. Lastly, menopausal status at first diagnosis was not associated with CBC risk.

**Table 2:** Age- and multivariable-adjusted risk ratios (RR) and 95 % confidence intervals (95 % CI) for associations of reproductive risk factors and contralateral breast cancer (CBC)

	Cases, N (%)	Controls, N (%)	Age-adjusted^a^ RR (95 % CI)	Multivariable^b^ RR (95 % CI)
Age at menarche (years)				
≤11	305 (20)	437 (20)	1.0 (ref.)	1.0 (ref.)
12	419 (28)	528 (24)	1.16 (0.93, 1.44)	1.16 (0.93, 1.44)
13	404 (27)	589 (27)	0.95 (0.77, 1.18)	0.96 (0.77, 1.19)
≥14	387 (25)	650 (29)	0.81 (0.65, 1.01)	0.82 (0.65, 1.03)
*P*_trend_^d^			*0.009*	*0.02*
Full-term pregnancies at first diagnosis				
None	311 (20)	399 (18)	1.0 (ref.)	1.0 (ref.)
≥1	1208 (79)	1810 (82)	0.91 (0.76, 1.08)	0.92 (0.77, 1.11)
Full-term pregnancies between first diagnosis and reference date				
Parous at first diagnosis, no interval pregnancy	1189 (78)	1785 (81)	1.0 (ref.)	1.0 (ref.)
Nulliparous at reference date	311 (20)	399 (18)	1.11 (0.93, 1.33)	0.96 (0.76, 1.21)
Full-term pregnancy between first diagnosis and reference date	19 (1)	25 (1)	1.30 (0.63, 2.66)	0.96 (0.76, 1.21)
Number of full-term pregnancies at first diagnosis^c^				
1	264 (22)	341 (19)	1.0 (ref.)	1.0 (ref.)
2	572 (47)	847 (47)	0.93 (0.74, 1.19)	0.97 (0.75, 1.25)
3	260 (22)	390 (22)	0.80 (0.61, 1.05)	0.87 (0.65, 1.17)
≥4	112 (9)	232 (13)	0.57 (0.41, 0.81)	0.60 (0.41, 0.88)
*P*_trend_^d^			*0.001*	*0.005*
Age at first full-term pregnancy at reference date (years)^c^				
<20	175 (14)	276 (15)	1.0 (ref.)	1.0 (ref.)
20–24	444 (37)	667 (37)	1.26 (0.97, 1.65)	1.19 (0.90, 1.58)
25–29	355 (29)	550 (30)	1.10 (0.83, 1.45)	1.01 (0.75, 1.35)
≥30	234 (19)	317 (18)	1.36 (0.99, 1.86)	1.20 (0.84, 1.70)
*P*_trend_^d^			*0.25*	*0.82*
Breastfeeding at reference date (months)^c^				
Never	680 (45)	940 (43)	1.0 (ref.)	1.0 (ref.)
Ever	837 (55)	1268 (57)	0.86 (0.75, 1.00)	0.84 (0.69, 1.02)
≤6	430 (28)	643 (29)	0.91 (0.77, 1.08)	0.88 (0.71, 1.09)
7–12	185 (12)	270 (12)	0.94 (0.74, 1.19)	0.91 (0.69, 1.19)
13–24	141 (9)	232 (10)	0.74 (0.58, 0.95)	0.72 (0.54, 0.96)
≥25	81 (5)	123 (6)	0.78 (0.56, 1.07)	0.77 (0.55, 1.10)
*P*_trend_^d^			*0.02*	*0.06*
Time since last full-term pregnancy, at reference date (years)^c^				
<5	37 (3)	50 (3)	1.12 (0.66, 1.90)	1.19 (0.66, 2.14)
5–<10	57 (5)	122 (7)	0.61 (0.41, 0.93)	0.63 (0.40, 1.01)
10 –<15	129 (11)	195 (11)	0.89 (0.65, 1.20)	0.93 (0.66, 1.31)
15–<20	203 (17)	305 (17)	0.96 (0.76, 1.21)	0.99 (0.76, 1.29)
≥20	782 (65)	1138 (63)	1.0 (ref.)	1.0 (ref.)
*P*_trend_^d^			*0.26*	*0.49*
Time between menarche and first full-term pregnancy at reference date (years)^c^				
<10	407 (34)	649 (36)	1.0 (ref.)	1.0 (ref.)
10–14	422 (35)	637 (35)	1.16 (0.94, 1.43)	1.20 (0.96, 1.49)
15–19	239 (20)	346 (19)	1.12 (0.87, 1.45)	1.10 (0.83, 1.44)
≥20	136 (11)	170 (9)	1.35 (0.97, 1.87)	1.33 (0.94, 1.88)
*P*_trend_^d^			*0.07*	*0.12*
Menopausal status/age at menopause at 2 years before first diagnosis				
Premenopausal	1124 (74)	1676 (76)	1.0 (ref.)	1.0 (ref.)
Postmenopausal/<45 years	195 (13)	282 (13)	1.10 (0.88, 1.38)	1.03 (0.81, 1.32)
Postmenopausal/≥45 years	194 (13)	240 (11)	1.27 (0.98, 1.64)	1.19 (0.91, 1.57)

^a^Including offset for countermatching

^b^Adjusted for age at first diagnosis, histology at first diagnosis, family history, stage at first diagnosis, chemotherapy/hormonal treatment for first diagnosis, use of postmenopausal hormone therapy up to first diagnosis, age at menarche, age at menopause and parity, with offset for countermatching

^c^Among parous women only, additionally mutually adjusted for age at first FTP, number of FTP, breast-feeding

^d^ *P*_trend_ calculated by modeling category medians continuously

Table 3 shows RRs for the associations of reproductive factors with CBC risk stratified by ER status of the first primary tumor. Among parous women, a history of breast-feeding was associated with a reduced risk of CBC risk only among women with ER-negative first tumors (ever breast-fed ER-negative vs. never breastfed RR = 0.69, 95 % CI 0.48–1.00 vs. ER-positive RR = 1.09, 95 % CI 1.09, 95 % CI 0.81, 1.47, *P*_heterogeneity_ = 0.05). Although there was no statistically significant trend observed with increasing duration, women with ER-negative tumors who had breast-fed for 25 months or longer had the greatest reduction in CBC risk relative to those who had never breast-fed (RR = 0.48, 95 % CI 0.22, 1.02, *P*_trend_ = 0.15). High parity was associated with reduced CBC risk regardless of ER status of the first tumor (*P*_heterogeneity_ = 0.95). Older age at FTP was associated with a lower risk of CBC among women with ER-negative first tumors, while a statistically non-significant positive association was observed among those with ER-positive disease (age at FTP ≥30 vs. <20 years ER-positive RR = 1.43, 95 % CI 0.87–2.36, *P*_trend_ = 0.18 vs. ER-negative RR = 0.66, 95 % CI 0.35–1.27, *P*_trend_ = 0.03; *P*_heterogeneity_ = 0.03). No statistically significant heterogeneity by ER status was observed for the time between menarche and first FTP; however, results indicate modest heterogeneity for longer intervals between menarche and FTP with risk increasing among women with ER-positive first tumors and risk decreasing among those with an ER-negative first tumor (*P*_heterogeneity_ = 0.10).

**Table 3:** Multivariable-adjusted risk ratios (RR) and 95 % confidence intervals (95 % CI) for associations of reproductive risk factors and contralateral breast cancer (CBC), by estrogen receptor (ER) status of first breast cancer

	ER-positive first tumors			ER-negative first tumors			*P*_heterogeneity_
	Cases, N (%)	Controls, N (%)	Multivariable^a^ RR (95 % CI)	Cases, N (%)	Controls, N (%)	Multivariable^a^ RR (95 % CI)	
Age at menarche (years)							
≤11	160 (20)	269 (21)	1.0 (ref.)	99 (21)	106 (19)	1.0 (ref.)	
12	226 (28)	301 (24)	1.25 (0.92, 1.70)	131 (28)	134 (24)	1.07 (0.70, 1.65)	
13	210 (26)	325 (26)	0.94 (0.69, 1.28)	121 (26)	151 (27)	1.04 (0.68, 1.59)	
≥14	199 (25)	358 (29)	0.93 (0.68, 1.28)	113 (24)	165 (29)	0.70 (0.45, 1.07)	
*P*_trend_^b^			*0.27*			*0.09*	*0.40*
Full-term pregnancies at first diagnosis							
None	173 (22)	248 (20)	1.0 (ref.)	95 (20)	98 (17)	1.0 (ref.)	
≥1	623 (78)	1004 (80)	0.93 (0.73, 1.19)	371 (79)	462 (82)	0.95 (0.65, 1.39)	*0.93*
Full-term pregnancies between first diagnosis and reference date							
Parous at first diagnosis, no interval pregnancy	620 (78)	990 (79)	1.0 (ref.)	361 (77)	455 (81)	1.0 (ref.)	
Nulliparous at reference date	173 (22)	248 (20)	0.97 (0.72, 1.30)	95 (20)	98 (17)	1.00 (0.66, 1.51)	
Full-term pregnancy between first diagnosis and reference date	3 (0)	14 (1)	0.21 (0.04, 1.16)	10 (2)	7 (1)	1.88 (0.57, 6.18)	*0.07*
Number of full-term pregnancies at first diagnosis^c^							
1	144 (23)	198 (20)	1.0 (ref.)	72 (19)	100 (22)	1.0 (ref.)	
2	289 (46)	467 (47)	1.05 (0.73, 1.51)	182 (49)	213 (46)	0.97 (0.60, 1.57)	
3	134 (22)	211 (21)	0.97 (0.64, 1.47)	82 (22)	91 (20)	0.98 (0.56, 1.71)	
≥4	56 (9)	128 (13)	0.52 (0.30, 0.90)	35 (9)	58 (13)	0.60 (0.30, 1.19)	
*P*_trend_^b^			*0.02*			*0.14*	*0.95*
Age at first full-term pregnancy at reference date (years)^c^							
<20	78 (13)	153 (15)	1.0 (ref.)	70 (19)	68 (15)	1.0 (ref.)	
20–24	211 (34)	359 (36)	1.14 (0.74, 1.76)	147 (40)	170 (37)	1.00 (0.59, 1.68)	
25–29	197 (32)	315 (31)	1.21 (0.77, 1.89)	91 (25)	132 (29)	0.56 (0.32, 0.99)	
≥30	137 (22)	177 (18)	1.43 (0.87, 2.36)	63 (17)	92 (20)	0.66 (0.35, 1.27)	
*P*_trend_^b^			*0.18*			*0.03*	*0.03*
Breastfeeding at reference date (months)^c^							
Never	180 (29)	308 (31)	1.0 (ref.)	136 (37)	150 (32)	1.0 (ref.)	
Ever	443 (71)	696 (69)	1.09 (0.81, 1.47)	233 (63)	311 (67)	0.69 (0.48, 1.00)	*0.05*
≤6	224 (36)	350 (35)	1.06 (0.76, 1.48)	111 (30)	172 (37)	0.65 (0.42, 1.00)	
7–12	82 (13)	148 (15)	1.03 (0.66, 1.61)	66 (18)	53 (11)	0.95 (0.55, 1.66)	
13–24	86 (14)	132 (13)	1.09 (0.69, 1.72)	35 (9)	50 (11)	0.83 (0.43, 1.62)	
≥25	51 (8)	66 (7)	1.53 (0.87, 2.72)	21 (6)	36 (8)	0.48 (0.22, 1.02)	
*P*_trend_^b^			*0.19*			*0.15*	*0.07*
Time since last full-term pregnancy, at reference date (years)^c, d^							
<15	104 (17)	250 (23)	0.87 (0.58, 1.32)	88 (24)	122 (26)	0.95 (0.59, 1.53)	
15–<20	104 (17)	165 (15)	1.06 (0.74, 1.54)	60 (16)	70 (15)	1.04 (0.65, 1.68)	
≥20	415 (67)	654 (61)	1.0 (ref.)	223 (60)	270 (58)	1.0 (ref.)	
*P*_trend_^b^			*0.65*			*0.74*	*0.99*
Time between menarche and first full-term pregnancy at reference date (years)^c^							
<10	191 (31)	358 (36)	1.0 (ref.)	141 (38)	156 (34)	1.0 (ref.)	
10–14	217 (35)	352 (35)	1.29 (0.93, 1.80)	128 (35)	164 (36)	0.88 (0.58, 1.34)	
15–19	131 (21)	192 (19)	1.38 (0.94, 2.05)	65 (18)	87 (19)	0.86 (0.51, 1.45)	
≥20	83 (13)	101 (10)	1.47 (0.93, 2.33)	35 (9)	50 (11)	0.58 (0.29, 1.17)	
*P*_trend_^b^			*0.06*			*0.15*	*0.10*
Menopausal status/age at menopause at 2 years before first diagnosis							
Premenopausal	583 (73)	932 (74)	1.0 (ref.)	348 (75)	345 (78)	1.0 (ref.)	
Postmenopausal/<45 years	99 (12)	164 (13)	1.00 (0.72, 1.40)	65 (14)	65 (12)	1.44 (0.92, 2.26)	
Postmenopausal/≥45 years	113 (14)	149 (12)	1.39 (0.98, 1.97)	50 (11)	59 (11)	1.03 (0.62, 1.71)	*0.19*

^a^Adjusted for age at first diagnosis, histology at first diagnosis, family history, stage at first diagnosis, chemotherapy/hormonal treatment for first diagnosis, use of postmenopausal hormone therapy up to first diagnosis, age at menarche, age at menopause and parity, with offset for countermatching

^b^Among parous women only, additionally mutually adjusted for age at first FTP, number of FTP, breastfeeding

^c^ *P*_trend_ calculated by modeling category medians continuously

^d^Categories collapsed due to small numbers

Results of analyses jointly stratified analyses by ER status of first tumor and menopausal status 2 years prior to first diagnosis are shown in Table 4. Older age at menarche was associated with reduced risk only among women who were postmenopausal at first diagnosis (≥14 vs. ≤11 years RR = 0.45, 95 % CI 0.25–0.81, *P*_trend_ < 0.001, *P*_heterogeneity_ = 0.0001), with similar RR estimates irrespective of ER status (Table 4). A history of breast-feeding was associated with reduced risk only among premenopausal women diagnosed with an ER-negative first tumor (*P*_heterogeneity_ = 0.07). Though tests of heterogeneity were not statistically significant, a long interval between menarche and first FTP was associated with an increased risk of CBC only among postmenopausal women who were diagnosed with an ER-positive first tumor (*P*_trend_ = 0.009), whereas among premenopausal women diagnosed with an ER-negative first tumor, a long interval was associated with reduced risk (*P*_trend_ = 0.05).

**Table 4:** Multivariable-adjusted risk ratios (RR) and 95 % confidence intervals (95 % CI) for associations of reproductive risk factors and contralateral breast cancer (CBC), by estrogen receptor (ER) status of first breast cancer and menopausal status 2 years prior to first breast cancer diagnosis

	ER-positive first tumors				ER-negative first tumors				P_*heterogeneity*_
	Premenopausal		Postmenopausal		Premenopausal		Postmenopausal		
	N, cases/controls	Multivariable^a^ RR (95 % CI)	N, cases/controls	Multivariable^a^ RR (95 % CI)	N, cases/controls	Multivariable^a^ RR (95 % CI)	N, cases/controls	Multivariable^a^ RR (95 % CI)	
Age at menarche (years)									
≤11	94/205	1.0 (ref.)	65/63	1.0 (ref.)	72/83	1.0 (ref.)	27/23	1.0 (ref.)	
12	166/219	1.74 (1.20, 2.53)	60/81	0.63 (0.36, 1.13)	95/108	0.96 (0.58, 1.58)	35/25	1.75 (0.71, 4.33)	
13	168/236	1.56 (1.08, 2.27)	41/85	0.29 (0.16, 0.52)	97/121	1.12 (0.69, 1.81)	23/30	0.75 (0.30, 1.86)	
≥14	153/271	1.33 (0.91, 1.93)	46/85	0.45 (0.25, 0.81)	81/120	0.79 (0.48, 1.32)	30/44	0.45 (0.20, 1.04)	
*P*_trend_^b^		*0.33*		*0.001*		*0.62*		*0.01*	*0.0001*
Full-term pregnancies at first diagnosis									
None	133/187	1.0 (ref.)	40/60	1.0 (ref.)	76/82	1.0 (ref.)	18/16	1.0 (ref.)	
≥1	449/744	0.87 (0.65, 1.15)	172/253	1.22 (0.74, 2.00)	272/353	0.97 (0.63, 1.48)	96/107	0.84 (0.36, 1.95)	*0.71*
Number of full-term pregnancies at first diagnosis^c^									
1	111/146	1.0 (ref.)	32/52	1.0 (ref.)	56/78	1.0 (ref.)	15/21	1.0 (ref.)	
2	221/369	0.99 (0.65, 1.49)	67/93	1.53 (0.74, 3.14)	140/175	1.02 (0.60, 1.74)	41/37	1.41 (0.41, 4.93)	
3	89/156	1.00 (0.62, 1.61)	45/53	1.22 (0.54, 2.75)	56/63	1.03 (0.53, 1.98)	25/28	1.57 (0.56, 4.42)	
≥4	28/73	0.41 (0.20, 0.82)	28/55	0.81 (0.34, 1.94)	20/37	0.64 (0.28, 1.44)	15/21	1.43 (0.44, 4.68)	
*P*_trend_^b^		*0.03*		*0.28*		*0.28*		*0.52*	*0.98*
Age at first full-term pregnancy at reference date (years)^c^									
<20	46/92	1.0 (ref.)	32/60	1.0 (ref.)	41/44	1.0 (ref.)	29/24	1.0 (ref.)	
20–24	136/252	1.12 (0.63, 1.96)	75/105	1.25 (0.64, 2.47)	104/117	1.14 (0.58, 2.21)	42/53	0.76 (0.32, 1.78)	
25–29	152/246	1.24 (0.70, 2.18)	44/68	1.38 (0.65, 2.95)	72/108	0.61 (0.31, 1.22)	18/23	0.51 (0.18, 1.47)	
≥30	115/154	1.35 (0.74, 2.47)	21/20	2.30 (0.84, 6.25)	55/84	0.64 (0.30, 1.41)	7/7	0.87 (0.19, 3.92)	
*P*_trend_^b^		*0.28*		*0.13*		*0.03*		*0.40*	*0.17*
Breastfeeding at reference date (months)^c^									
Never	111/198	1.0 (ref.)	69/106	1.0 (ref.)	98/106	1.0 (ref.)	38/43	1.0 (ref.)	
Ever	338/546	1.26 (0.87, 1.81)	103/147	0.90 (0.52, 1.54)	172/246	0.62 (0.40, 0.96)	58/64	0.72 (0.34, 1.51)	*0.07*
≤6	164/259	1.31 (0.87, 1.97)	58/89	0.74 (0.40, 1.36)	75/127	0.59 (0.35, 1.00)	34/44	0.59 (0.25, 1.38)	
7–12	63/118	1.10 (0.65, 1.87)	19/29	1.13 (0.48, 2.63)	51/43	0.94 (0.49, 1.79)	14/10	0.83 (0.26, 2.67)	
13–24	72/112	1.24 (0.74, 2.08)	14/20	1.33 (0.41, 4.35)	28/46	0.71 (0.35, 1.48)	7/4	1.59 (0.23, 10.8)	
≥25	39/57	1.78 (0.93, 3.38)	12/9	1.50 (0.37, 6.04)	18/30	0.37 (0.16, 0.86)	3/6	1.23 (0.17, 9.17)	
*P*_trend_^b^		*0.20*		*0.28*		*0.06*		*0.71*	*0.19*
Time between menarche and first full-term pregnancy at reference date (years)^c^									
<10	119/237	1.0 (ref.)	72/118	1.0 (ref.)	90/102	1.0 (ref.)	50/54	1.0 (ref.)	
10–14	161/260	1.26 (0.85, 1.870	56/91	1.52 (0.82, 2.81)	96/121	0.86 (0.51, 1.44)	31/42	0.91 (0.41, 1.99)	
15–19	103/160	1.32 (0.84, 2.06)	27/31	1.85 (0.84, 4.04)	56/81	0.72 (0.40, 1.29)	9/6	1.74 (0.39, 7.81)	
≥20	65/86	1.25 (0.74, 2.08)	17/13	3.70 (1.27, 10.9)	28/46	0.46 (0.21, 1.01)	6/3	1.42 (0.26, 7.93)	
*P*_trend_^b^		*0.30*		*0.009*		*0.05*		*0.59*	*0.17*

^a^Adjusted for age at first diagnosis, histology at first diagnosis, family history, stage at first diagnosis, chemotherapy/hormonal treatment for first diagnosis, use of postmenopausal hormone therapy up to first diagnosis, age at menarche, age at menopause and parity, with offset for countermatching

^b^Among parous women only, additionally mutually adjusted for age at first FTP, number of FTP, breastfeeding

^c^ *P*_trend_ calculated by modeling category medians continuously. Estimates for time since last full-term pregnancy and interval pregnancies not shown due to small numbers

## Discussion

Results from our large, population-based case-control study indicate that age at menarche and parity are related to CBC risk. Consistent with our findings from the first phase of the WECARE Study (Largent et al. 2007), we observed inverse associations of increasing age at menarche and higher parity with CBC risk in our expanded study population. Additionally, the risk varied by ER status of the first primary tumor and menopausal status at first diagnosis, suggesting etiologic differences across tumor subtype. For example, breast-feeding was associated with a reduced CBC risk only among women with ER-negative tumors and was limited to women who were premenopausal at first diagnosis. The inverse association between age at menarche and CBC risk was observed only among women who were postmenopausal at first diagnosis, regardless of ER status of the first cancer. Long interval between menarche and first FTP was positively associated with CBC risk only among postmenopausal women with an ER-positive first tumor.

Previous studies of reproductive factors and CBC risk have produced somewhat inconsistent results, and many were limited by small sample sizes and limited covariate data. Excepting our prior analysis (Largent et al. 2007), studies of U.S. women have included fewer than 400 CBC cases each (Bernstein et al. 1992; Boice et al. 1992; Cook et al. 1996; Horn and Thompson 1988; Li et al. 2003). Studies conducted within European cancer registries have had larger sample sizes. The largest to date had 2529 CBC cases with limited reproductive data identified in the Swedish Family-Cancer Database (Vaittinen and Hemminki 2000), and reported modest inverse associations for both parity and age at first birth with CBC risk; however, no other reproductive risk factors were examined in this study. Most recently, in an analysis including 121 CBC cases in the European Investigation into Cancer and Nutrition (EPIC) Study, Ricceri et al. (2015) reported a 42 % reduced risk of CBC for parous relative to nulliparous women; however, data on important covariates, including mastectomy, were not available.

Our data suggest that the importance of early hormonal and cellular changes mediated through reproductive events persists across multiple cancer events. Following a first FTP, risk of developing breast cancer is elevated; however, risk decreases over time, leading to a net protective influence of pregnancy on lifetime risk for women with younger ages at first FTP relative to nulliparous women (Rosner et al. 1994; Trichopoulos et al. 1983; Albrektsen et al. 2005; Hsieh et al. 1994; Lambe et al. 1994). The protective effect of parity on breast cancer risk may be mediated through pregnancy-induced differentiation of mammary stem cells, which reduces the population of cells at risk for malignant transformation (Russo et al. 1982). While we observed an inverse association between number of FTP and CBC risk, age at first FTP was not associated with risk. In contrast, we observed that CBC risk was suggestively elevated among women with long intervals between menarche and first FTP. It is possible that this interval has greater biologic relevance to breast cancer than age at first FTP alone, as it may more accurately reflect the period during which rapidly expanding, undifferentiated breast epithelium is most vulnerable to carcinogenesis (Pike et al. 1983; Li et al. 2008). Age at menarche itself was also inversely associated with CBC risk, consistent with findings for primary breast tumors (Collaborative Group on Hormonal Factors in Breast Cancer 2012).

To date, ours is the first analysis to examine associations between reproductive factors and CBC risk according to ER status of the first primary tumor. We observed that a history of breast-feeding appeared to reduce risk of CBC among women diagnosed with an ER-negative first breast cancer; women who breast-fed for 25 months or more had the greatest reduction in risk, though no significant trend was observed. Women with ER-negative first tumors have higher CBC risk (Saltzman et al. 2012; Kurian et al. 2009) and do not benefit from treatment with tamoxifen (Early Breast Cancer Trialists’ Collaborative Group et al. 2011). Breast-feeding has consistently been associated with reduced risk of first primary ER-negative subtypes (Gaudet et al. 2011; Millikan et al. 2008; Tamimi et al. 2012; Palmer 2014). Some evidence has suggested that breast-feeding may reduce breast cancer risk even among women at highest risk of breast cancer, specifically *BRCA1* mutation carriers (Jernstrom et al. 2004; Kotsopoulos et al. 2012), who tend to develop ER-negative tumors (Foulkes et al. 2004) and have elevated CBC risk (Graeser et al. 2009; Malone et al. 2010). In the present study, we lacked *BRCA1* and *BRCA2* mutation status for women sampled in WECARE II; however, our results suggest that breast-feeding may mitigate some of the increased risk of CBC associated with having an ER-negative first primary tumor.

We additionally observed evidence of heterogeneity in the associations of age at first FTP with CBC risk by ER status of the first tumor. Older age at first FTP was suggestively associated with increased risk among women with ER-positive first tumors, but inversely associated with CBC risk among women with ER-negative first tumors. These findings are consistent with a recent review (Anderson et al. 2014), which indicated that late age at first pregnancy was positively associated with risk of hormone receptor-positive first primary tumors in 15 out of 22 published analyses, while associations with hormone receptor-negative tumors reported from 13 analyses were null or inverse. Though no statistically significant heterogeneity was observed, a long interval between menarche and first FTP was associated with an increased CBC risk among women with ER-positive first breast cancers only, consistent with findings from some previous analyses of first primaries (Ritte et al. 2013; Li et al. 2008; Chung et al. 2013; Ambrosone 2015). Here, we did not observe heterogeneity by ER status for the association between age at menarche and CBC risk, though a suggestive inverse association was observed for ER-negative tumors only. Instead, our results suggest that the effects of menarcheal age on CBC risk may be modified by the hormonal milieu, with stronger inverse associations observed for women who were postmenopausal at first diagnosis, regardless of ER status. However, in a large meta-analysis (Collaborative Group on Hormonal Factors in Breast Cancer 2012), younger age at menarche was associated with risk of both pre- and postmenopausal primary breast cancer, with a weakening of the effect among postmenopausal women observed with increasing age at diagnosis.

Some limitations of our study must be acknowledged. Although our study benefited from a large sample size, only 44 FTPs occurred after first diagnosis, and we therefore had limited power to examine whether pregnancy or breast-feeding following a first primary breast cancer diagnosis is associated with CBC risk. Small numbers for some subgroups (i.e., ER-negative tumors/postmenopausal status) may have also limited our ability to detect heterogeneity in some stratified analyses. In order to qualify for inclusion into our study, participants had to be alive at time of recruitment; we cannot rule out the possibility that women who died before this time had different distributions of reproductive factors than those who were alive. In order to limit the potential for resulting survival bias, we restricted eligibility to women whose cancer had not spread beyond regional lymph nodes, and matched cases and controls on both age and year categories of first diagnosis.

Our results provide further evidence that some reproductive factors known to be associated with first primary breast cancers are additionally associated with CBC risk. Specifically, we confirmed our previous findings from the WECARE I study population, which indicated that older age at menarche and increasing parity appear protective for CBC. While not associated with overall CBC risk, increasing age at first FTP appeared inversely associated with risk among women with ER-negative first primaries. Additionally, while we found no association between breast-feeding and CBC risk our previous study, here, we report an inverse association that appears limited to women with ER-negative first tumors, who are at a higher risk of CBC, particularly ER-negative subtypes [Sisti et al., submitted]. These results add to others which suggest that breast-feeding may play a protective role in the etiology of ER-negative breast cancers, and provide the first evidence that this protective effect persists for second breast tumors. Further studies are needed to clarify these associations.
